# Sudden Vision Loss Due to Optic Neuritis—An Uncommon Presentation of Neurosarcoidosis

**DOI:** 10.3390/diagnostics13152579

**Published:** 2023-08-03

**Authors:** Katarzyna Zimna, Monika Szturmowicz, Małgorzata Sobiecka, Katarzyna Błasińska, Małgorzata Bartosiewicz, Witold Z. Tomkowski

**Affiliations:** 1 1st Department of Lung Diseases, National Tuberculosis and Lung Diseases Research Institute, 01-138 Warsaw, Poland; 2 Department of Radiology, National Tuberculosis and Lung Diseases Research Institute, 01-138 Warsaw, Poland

**Keywords:** sarcoidosis, optic nerve neuritis, neuro-sarcoidosis

## Abstract

Sarcoidosis is a systemic, granulomatous disease of unknown etiology, most often manifested by mediastinal and hilar lymph node enlargement and parenchymal nodules in the lungs. However, it may involve any other organ. Neuro-sarcoidosis, a condition that affects up to 20% of sarcoidosis patients, can be found in any part of the central or peripheral nervous system and has important ophthalmic and neuro-ophthalmic manifestations. We present two patients with sudden vision loss due to neurosarcoidosis. In both cases, biopsy of the mediastinal lymph node showed non-caseating granulomas consistent with sarcoidosis. Treatment involved high doses of methylprednisolone intravenously, followed by topical dexamethasone eye drops in the first case and a systemic steroid treatment in the second, resulting in symptom relief. Those two cases demonstrate that sarcoidosis should be considered as a differential diagnosis in cases of optic neuritis.

## 1. Introduction

Sarcoidosis is a systemic, granulomatous disease of unknown etiology, most often manifested by mediastinal and hilar lymph node enlargement and parenchymal nodules in the lungs. However, it may affect any other organ [1]. Neurosarcoidosis is recognized in 5–20% of sarcoidosis patients [2,3]. The most often-described neurological manifestations include: cranial nerve involvement (facial nerve palsy, less often optic neuritis and oculomotor nerve neuritis), small fiber neuropathy (causing severe pain and vegetative system disorders), less often—meningitis, spinal cord involvement, brain lesions and disorders of the pituitary-hypothalamic axis [4,5,6]. Optic neuropathy may occur in 33–70% of cases of neuro-ophthalmic sarcoidosis and may manifest as optic neuritis (retrobulbar or with papillitis), optic atrophy, papilledema or optic nerve granuloma. One third of the patients with optic nerve involvement develop severe visual deficiency [6,7]. More than 80% of patients with neurosarcoidosis have associated systemic sarcoidosis (mainly in the lungs and lymph nodes) [8].

The most challenging manifestation of neurosarcoidosis is sudden vision loss, especially if it is the first manifestation of sarcoidosis. We describe two patients with this uncommon complication.

## 2. Case Report 1

**Figure 1:**
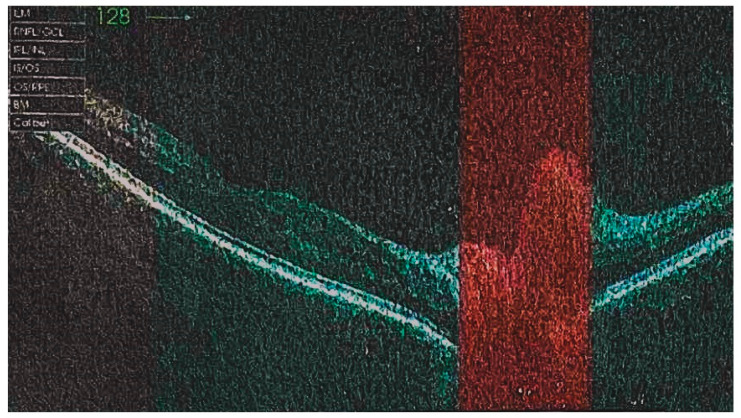
Optical coherence tomography of the right eye showing edema of the optic disc.

**Figure 2:**
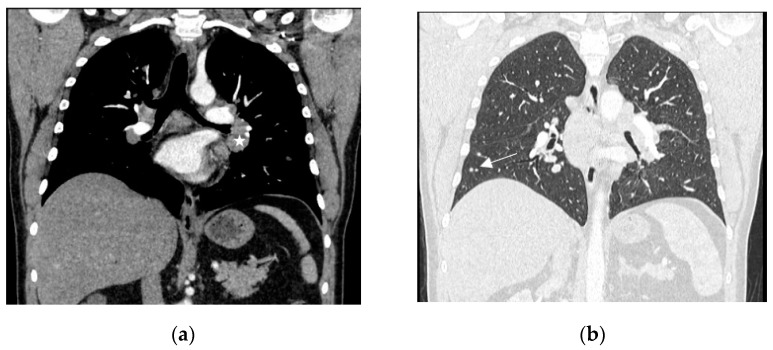
Computed tomography of the chest, with contrast enhancement, coronal plane, mediastinal window (**a**), lung window (**b**). Enlarged mediastinal and hilar lymph nodes ((**a**)—white asterisk). Small nodules in the right lower lobe ((**b**)—white arrow).

A 44-year-old patient, non-smoker, presented himself at the emergency department elsewhere due to sudden onset of a vision loss in the right eye. He had a history of arterial hypertension, hyperlipidaemia and depression. The ophthalmological examination revealed oedema of the right optic nerve with narrowed peripheral vision. No signs of uveitis were found. The optical coherence tomography (OCT) demonstrated right papilledema (Figure 1). The clinical neurological examination, except for the vision limitation, was unremarkable and computed tomography (CT) of the central nervous system was unexceptional. Infectious causes such as Herpes simplex virus (HSV), Toxoplasma gondii, Cytomegalovirus (CMV), Lyme disease, Hepatitis C virus (HCV), Human immunodeficiency virus (HIV), Hepatitis B virus (HBV) were also excluded. Otherwise, CRP (C- reactive protein), anti-nuclear antibodies (ANA) and rheumatoid factor (RF) were negative and serum angiotensin converting enzyme (ACE) level was normal. In the performed chest X-ray and consecutive chest CT, enlarged mediastinal and hilar lymph nodes with parenchymal micronodules have been described. The patient was treated with a high-dose of intravenous methylprednisolone (500 mg/day) for three days, followed only by topical dexamethasone drops, resulting in rapid symptoms relief. The suspicion of sarcoidosis was established and the patient was referred to our department in order to evaluate pulmonary lymphadenopathy. There was a two-month delay for non-substantive reasons, before he presented himself to our department. Before admission, he underwent an ophthalmologic examination, which showed no abnormalities and the topical treatment was terminated. He reported a gradual decrease in exercise tolerance for about 3 years, which was not further diagnosed and denied other respiratory symptoms. His laboratory test showed slightly elevated serum glucose to 109 mg% and N-terminal prohormone of brain natriuretic peptide (NT-proBNP) to 132 pg/mL (normal: <125 pg/mL). Calcium concentration in serum and in daily urine collection was within normal limits. Chest X-ray, performed at the time of admission, showed symmetrical, polycyclic hilar dilatation. Chest CT revealed numerous moderately-enlarged lymph nodes in the mediastinum (subcarinal 30 × 38 mm, prevascular 17 mm, left hilum up to 18 mm, right hilum up to 20 mm) and parenchymal changes including bilateral peri-lymphatic micronodules (Figure 2). Compared to the previous chest CT scan, partial regression of disseminated lung lesions was visible, lymphadenopathy appeared to be stable. The patient was consulted by an ophthalmologist and normal optic nerve discs were found on both sides. On the periphery of the retina, “cobblestones” were visible—without degenerative changes predisposing to retinal detachment. Abdominal ultrasonography revealed liver steatosis. Pulmonary function tests were within the normal range. Echocardiography (ECHO) showed mild left ventricular wall hypertrophy and discrete hypokinesia of the central segment of the interventricular septum (IVS). Hence, magnetic resonance imaging (MRI) of the heart was performed, which showed no radiological features of active myocarditis, left ventricular ejection fraction was within normal limits. In order to confirm the diagnosis, a bronchoscopy with bronchial mucosa biopsy, bronchoalveolar lavage (BAL) and endobronchial ultrasound-guided transbronchial needle aspiration (EBUS TBNA) of the subcarinal lymph nodes were performed. Histopathological examination revealed fragments of the bronchial mucosa with non-necrotizing granulomas. Cultures of the bronchial fluid were negative for bacterial, fungal and mycobacterial pathogens. Based on the results, a diagnosis of sarcoidosis with optic neuritis was established. According to the European Respiratory Society (ERS) guidelines, oral glucocorticoid treatment should have been started after high-dose of intravenous methylprednisolone therapy, nevertheless the patient received only the topical treatment. We speculated on whether the oral glucocorticoid treatment should be initiated after two months of a delay. However, at the time of admission to our department, two months apart from the onset of optic neuritis, no activity of the disease had been confirmed, hence further observation was recommended. In a follow-up chest X-ray conducted after three months (which equals 5 months after the onset of the optic neuritis), a regression of the hilar lymph nodes was observed. His ophthalmological examination and laboratory test showed no abnormalities. He was scheduled for strict regular follow-up appointments.

## 3. Case Report 2

**Figure 3:**
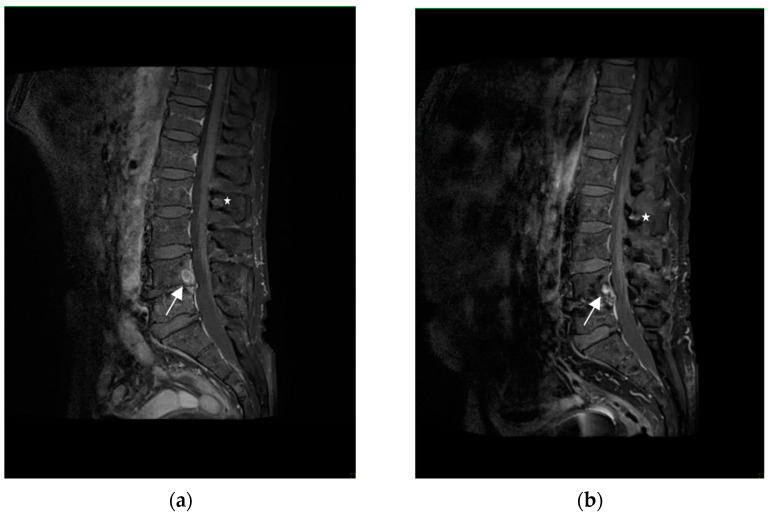
T1-weighted images with fat saturation and with gadolinium contrast administration, sagittal plane. Contrast enhanced lesion in L4 vertebral body ((**a**)—white arrow) has reduced its size ((**b**)—white arrow). Similarly, the lesion in the spinous process L2 ((**a**)—white asterisk) has decreased in size ((**b**)—white asterisk).

**Figure 4:**
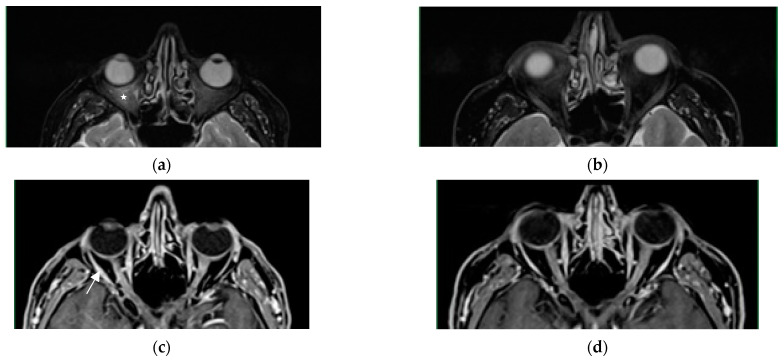
T2-weighted images with fat saturation, axial plane—(**a**,**b**). Moderate edema of the intraconal fat secondary to optic neuritis ((**a**)—white asterisk). Resolution of edema on follow—up examination (**b**). T1-weighted image with gadolinium contrast administration, axial plane—(**c**,**d**). Heterogeneous optic nerve enhancement, more significant on the right side ((**c**)—white arrow). Marked partial regression of lesions on follow-up examination (**d**).

A 63-year-old man with a history of benign prostatic hyperplasia, an ex-smoker with a history of 15 pack-years, presented himself to the general practitioner due to sudden onset of severe chest pain, that radiated to his back, the interscapular region, and the lumbosacral area, as well as circumferential pain around the subcostal region. The pain did not respond to any painkillers. The patient reported weight loss of 12 kg in the past 6 months due to reduced appetite, but denied cough, dyspnoea, haemoptysis and fever. Physical examination did not reveal any significant abnormalities. Laboratory tests showed a slightly elevated erythrocyte sedimentation rate (ESR) of 40 mm/h (normal: 3–8 mm/h) and a CRP level of 36 mg/L (normal: <5 mg/L), but were otherwise within normal limits. ANA and RF were negative. A chest CT revealed multiple enlarged mediastinal and hilar lymph nodes (up to 44 × 21 mm in diameter), as well as a 10 mm nodule in segment 6 of the left lung. Abdominal and pelvic CT showed several hypodense, small (4–6 mm) focal lesions in the liver, which were nonspecific, and a few nonspecific hypodense areas of bone rarefaction up to 6 mm in size in the lumbar vertebrae. Histological examination of the mediastinal lymph node (group 7) obtained by EBUS-TBNA showed non-necrotizing granulomas and confirmed the diagnosis of sarcoidosis. To assess the disease extent, a positron emission tomography (PET)-CT scan was performed, which revealed numerous metabolically-active lymph nodes symmetrically distributed in the mediastinum and hilar regions, as well as a few metabolically active and inactive nodules in the lungs, which were most likely granulomatous in nature, but no metabolically active lesions were found in the abdominal organs or bones. An MRI of the thoracic and lumbar spine showed four nonspecific intramedullary foci in the L2 and L4 vertebral bodies, and the Th9 and Th11 thoracic vertebrae, which enhanced peripherally with contrast, and could represent either atypical hemangiomas or sarcoid granulomas, but their nature could not be definitively determined at that time (Figure 3). The patient was evaluated by a neurologist, who did not find any signs of brain or spinal cord damage, nor the cranial nerve or root involvement. Abdominal ultrasonography and ECHO were performed, and no abnormalities were detected. The parameters of the pulmonary function tests were normal. The serum calcium concentration and its daily excretion in urine were normal. Based on all the tests and clinical course, stage II sarcoidosis was diagnosed. Three weeks after the completion of the diagnostic workup, sudden loss of vision in the right eye occurred, and the patient was hospitalized in the ophthalmology department, where optic neuritis was diagnosed and intravenous methylprednisolone (1000 mg/day) for four days was initiated, followed by oral prednisone (60 mg/day). An MRI examination of the orbits confirmed the presence of lesions suggestive of optic neuritis (visual nerves with slightly uneven bilateral enhancement of the intraorbital parts, focal enhancement of the optic chiasm and left optic tract, and mild periorbital fat swelling of the right eye). No deviations were found in the MRI of the central nervous system (Figure 4). After steroid treatment was initiated, the patient regained vision, although there was still a limitation of the visual field in the right eye (which remained stable during further observation). He also reported a complete chest pain relief. In the follow-up MRI examination of the orbits, the periorbital fat swelling of the right eye subsided compared to the previous examination and there was no focal enhancement of the optic chiasm and left optic tract. In the follow-up MRI examination of the lumbar and thoracic spine, the two intramedullary foci in L2 and L5 decreased, the focus in Th11 regressed completely, and the one In Th9 remained stable—considering the dynamics of changes in vertebral bodies and response to the steroid treatment, involvement of bone marrow in sarcoidosis was confirmed. Prednisone treatment in tapered dose was continued for 2 years.

Three years after the termination of the steroid treatment, the patient began to complain of progressive weakness. A CT of the chest confirmed the disease progression in the lungs. Normocytic anemia, hypercalcemia and a decrease in the estimated glomerular filtration rate (eGFR) were found in laboratory tests. The parameters of the pulmonary function tests were normal as previously. Echocardiography did not reveal any features suggestive of cardiac sarcoidosis or significant pulmonary hypertension. The loss of visual field in the right eye remained stable—the patient remained under the care of the ophthalmologist. Based on the overall clinical-radiological picture and additional diagnostic tests, a relapse of sarcoidosis with hypercalcemia and renal impairment was diagnosed. Prednisone was re-initiated with a dose of 40 mg/day. After one week of treatment, serum calcium levels normalized. The prednisone dose was gradually reduced to 10 mg/day. Partial regression of diffuse and nodular changes was observed in radiological imaging, with a significant increase in eGFR and resolution of anemia in laboratory tests. Since the onset of illness, the patient denied respiratory symptoms, and pulmonary function tests did not show any ventilatory abnormalities.

## 4. Discussion

The two abovementioned case reports describe patients with sarcoidosis complicated by optic neuritis. Neuro-sarcoidosis, a condition that affects up to 20% of sarcoidosis patients, can be found in any part of the central or peripheral nervous system and has important ophthalmic and neuro-ophthalmic manifestations [9,10,11]. Most commonly, sarcoidosis affects the uveal tract. Nevertheless, the optic nerve is also commonly involved. Various mechanisms can cause vision loss in optic nerve involvement, such as granulomatous infiltration, compression, or increased intracranial pressure. Typical clinical signs of optic neuritis associated with sarcoidosis include subacute or sudden onset of moderate to severe vision loss in the course of unilateral papilledema. Cranial MRI presents hyperintensity on T2 weighted images with contrast enhancement and the presence of granulomas along the optic nerve [10,12]. Due to unremarkable cranial CT scans, cerebral MRI in the first patient was not performed at the onset of the symptoms, hence there was no evaluation of the probable lesions on the optic nerve itself nor the sheath. In the second patient, a cranial MRI showed signs of an optic neuritis. Sarcoidosis-related optic nerve involvement usually results in poor visual outcomes and a high rate of early relapse [3,13]. In the first case, optic neuritis with sudden onset was the first sign of sarcoidosis. The diagnosis was confirmed by finding non-caseating granulomas in the bronchial mucosa biopsy. The second patient had already been diagnosed with a sarcoidosis, before the onset of sudden vision loss. Another sign of neurosarcoidosis was the small fiber neuropathy.

The use of OCT has significantly improved the detection and treatment of retinal and choroidal disease. In sarcoidosis cases, OCT can provide clinicians with an improved ability to differentiate between subretinal and choroidal lesions, leading to more accurate differential diagnoses. Enhanced depth imaging (EDI), a special feature in modern OCT, allows for Bruch’s membrane penetration and enhances the resolution of the choroid [10]. In the first case, OCT demonstrated right papilledema. The second patient did not undergo such a test.

It is essential to distinguish between neurosarcoidosis and demyelinating optic neuritis, which is linked to multiple sclerosis (MS), especially in younger patients. Bilateral vision loss or features suggestive of chiasmal involvement may indicate demyelinating optic neuritis, rather than sarcoid-related optic neuropathy, which rarely presents as bilateral or chiasmal disease [10,14]. Choroidal granulomas are considered to be one of the retinal abnormalities indicating granulomatous processes found in infectious diseases such as tuberculosis or non-infectious, such as sarcoidosis [14,15]. Non-specific findings on visual field testing include caecocentral scotomas, enlarged blind spots, reduced ocular deviation, and hemifield loss [14]. In one case series, the most frequent sign of neurosarcoidosis associated with optic neuropathy was optic disc oedema [15]. Optic perineuritis, which involves optic nerve sheath inflammation, is considered to be another sign of neurosarcoidosis, associated with optic disc oedema, observed in ophthalmological examination. Typically, the central vision is impaired, although patients may show other signs of optic nerve malfunction, which include decreased peripheral vision or dyschromatopsia. [10]. Another less common, but highly indicative manifestation of sarcoidosis-related optic involvement, is optic disc granuloma. In patients with detected bilateral optic nerve oedema and its impaired function, elevated intracranial pressure must be excluded as the primary cause. Obstructive hydrocephalus may be a result of sarcoid granulomas affecting the meninges or brain lesion. Involvement of the basal leptomeninges is another manifestation of neurosarcoidosis, which can lead to optic disc swelling [10].

The diagnosis of sarcoidosis at this date is not standardized. According to the American Thoracic Society (ATS), the diagnostic pathway relies on three main criteria: a suitable clinical manifestation, the presence of non-necrotizing granulomatous inflammation in one or several samples of tissue (which is not always necessary), and ruling out other potential causes of granulomatous disease [16]. The presented patients met all three major criteria. A common biopsy sample may be retrieved from the lungs, lymph nodes, skin, conjunctivae, lacrimal glands or orbital tissues. However, biopsy may not be feasible or desirable for some patients, especially if suspected lesions are not easily accessible. Radiological imaging such as chest X-ray, chest CT scan, and magnetic resonance imaging (MRI) may be helpful in aiding the diagnosis. Regarding neurosarcoidosis, brain, and spinal MRI with gadolinium enhancement is considered as “gold standard” and should be used in diagnostic evaluation and for radiological follow-up [8]. Affected cranial nerves appear hyperintense with thickening of the nerve dura on MRI scans [6].

Large-scale randomized controlled trials (RCTs) have not been conducted to thoroughly investigate the effectiveness of any of the medications currently applied for the treatment of sarcoidosis [1,2,17]. By providing potential treatment strategies for different organ manifestations of sarcoidosis, the newly released clinical practice guidelines from the ERS aim to tackle these issues [2,18]. The guidelines propose using glucocorticoids to treat clinically significant neuro-sarcoidosis, with additional second-line medications, such as methotrexate, azathioprine (AZA), mycophenolate mofetil (MMF), antimalarial drugs, or cyclosporine A, used concurrently with glucocorticoids for initial treatment. Although the data are limited, methotrexate and hydroxychloroquine are believed to be more effective than mycophenolate mofetil or azathioprine in reducing the relapse rate of neuro-sarcoidosis [3]. If there is a threat of vision loss, high dose intravenous steroid therapy should be initiated and followed by an oral glucocorticoid treatment. The initial treatment should last for 3–6 months with dose reduction to the minimal effective dose daily. Methotrexate is the preferred second-line agent for neuro-sarcoidosis, according to the guidelines. In cases where neuro-sarcoidosis is still active or relapses while on glucocorticoids and a second-line agent, Tumor necrosis factor (TNF)-alpha inhibitor- infliximab is the preferred medication, and adalimumab is recommended as an alternative to infliximab [2,18,19]. The guidelines of the Delhi Consensus recommend early or simultaneous use of non-biologic "second-line" agents, such as steroid-sparing non-biologic immunosuppressive therapy, in patients with severe or multi-organ disease or those at high risk of steroid-induced toxicity [20]. Optic neuritis is a severe manifestation of a neurosacroidosis, and determining whether or not steroid treatment along with a second line therapy (methotrexate) should be introduced earlier in the therapeutic strategy must be considered. In a recent literature review, the proportion of a favorable outcome in patients with neurosarcoidosis treated with TNF-alpha inhibitor was higher, compared to methotrexate, MMF and AZA (*p* < 0.00001) [8]. In both of our patients, intravenous treatment with a high dose of methylprednisolone had resulted in partial regression of the optic neuritis and pulmonary disease. The first patient did not undergo an oral steroid treatment, due to a diagnostic delay and lack of evidence of any organ malfunction at time of our clinical evaluation. Topical dexamethasone treatment was terminated after two months by an ophthalmologist and the patient was sustained under strict follow-up examinations, with no relapse after 5 months of observation. Although this treatment was not confluent with ERS guidelines, the question arises whether high-dose intravenous glucocorticoid treatment followed by topical treatment may be sufficient in neurosarcoidosis limited to optic neuritis. This issue requires further research on a specially selected group of patients. The second patient received oral prednisone therapy following intravenous glucocorticoid pulses. Both optic neuritis and small fiber neuropathy subsided during treatment. The patient experienced relapse of the disease three years after the prednisolone therapy was terminated, which manifested with hypercalcemia and renal impairment. Nevertheless, ophthalmic involvement was stable, with no signs of disease progression in the eye. Therefore, prolonged prednisone therapy was decided.

In sarcoidosis, numerous randomized controlled trials (RCTs) are currently in progress to assess the efficacy of new therapeutic compounds, innovative treatment targets, and alternatives to steroid treatment. Several of these trials have produced promising results in the initial phases [1].

## 5. Conclusions

Sarcoidosis-related neuro-ophthalmic manifestations can include the orbital region, afferent and efferent pathways, and all components of the visual system. Isolated optic nerve involvement is insufficient to diagnose sarcoidosis, and other differential diagnoses such as meningioma or glioma should be excluded. In cases of optic neuritis, ophthalmologists should consult with other specialists and consider sarcoidosis as a potential diagnosis, if there is mediastinal and bilateral hilar lymphadenopathy [13]. Ophthalmic signs may be clinically silent, hence patients with newly diagnosed sarcoidosis should undergo a routine eye examination [16].

## Data Availability

Not applicable.
